# Inhibition of HSP90 sensitizes a novel Raf/ERK dual inhibitor CY-9d in triple-negative breast cancer cells

**DOI:** 10.18632/oncotarget.22119

**Published:** 2017-10-26

**Authors:** Yujuan Chen, Xiaoyun Wang, Chuan Cao, Xiaodong Wang, Shufang Liang, Cheng Peng, Leilei Fu, Gu He

**Affiliations:** ^1^ State Key Laboratory of Biotherapy and Department of Breast Surgery, West China Hospital, West China Medical School, Sichuan University and Collaborative Innovation Center for Biotherapy, Chengdu 610041, China; ^2^ State Key Laboratory Breeding Base of Systematic Research Development and Utilization of Chinese Medicine Resources, School of Pharmacy, Chengdu University of Traditional Chinese Medicine, Chengdu 611137, China

**Keywords:** breast cancer, Raf, ERK, HSP90, apoptosis

## Abstract

Raf and extracellular signal-regulated kinases (ERK) are both important therapeutic targets in the mitogen-activated protein kinase (MAPK) pathway, and play crucial roles in the apoptosis resistance of breast cancer cells. In the present study, cytotoxic and apoptosis-inducing activities of the Raf/ERK dual inhibitor CY-9d were found to be restricted in triple negative breast cancer (TNBC) cells compared with ER/PR-positive cells. Based on the analysis of differentially expressed proteins using a quantitative proteomic iTRAQ method and bioinformatics analysis, HSP90 was found to identify as a potential mediator between Raf and ERK in TNBC cells. Western blotting and RNA interference suggested that down-regulated IQGAP1 can attenuate the routine Raf/MEK/ERK cascade and recruit HSP90 as a bypass pathway. Simultaneous treatment with the HSP90 inhibitor and CY-9d at sub-therapeutic doses was found to produce synergistic therapeutic and apoptosis-inducing effects in TNBC cells. Moreover, CY-9d was also found to suppress breast cancer growth, inhibit the activation of Raf/ERK, and induce mitochondrial apoptosis *in vivo* without remarkable toxicity. These results support the combination of HSP90 and Raf/ERK inhibitors as a potential target therapeutic strategy with enhanced tumor growth suppression, downstream pathway blockade, and greater induction of apoptosis.

## INTRODUCTION

According to cancer statistics published in 2017, breast cancer accounts for approximately 30% of female cancer cases, and after lung cancer, is the second cancer-related mortality in females worldwide [1]. Numerous studies have suggested that hyper-activation of the MAPK signaling pathway is common in breast cancer, and therapies targeting MAP kinases have played important roles in the treatment of breast cancer [2-11]. In the MAPK signaling pathways, the Raf/MEK/ERK cascade is activated by mutant KRAS and plays a crucial role in the regulation of cancer cell proliferation, differentiation, survival and migration [12-15]. The oncoprotein Ras is one of the main regulators of receptor tyrosine kinase-induced cell proliferation and survival in both normal and cancerous cells. Activated Ras can phosphorylate proteins in the Raf/MEK/ERK, PI3K/AKT/mTOR and other signaling pathways [16-19]. As a direct downstream substrate of Ras, Raf activation leads to the phosphorylation of MEK, the kinase that activates ERK. ERK regulates the phosphorylation of P90RSK and resulting nuclear transcriptional changes that alter cell survival, migration and angiogenesis [18-24]. Many research teams are attempting to discover agents that directly target Raf-MEK-ERK pathways [25-31]. Raf or MEK inhibition yields good responses in some BRAF-mutant or KRAS-mutant cancers, but some patients have not been found to benefit from a single-target therapeutic approach [16]. Several research groups have attempted to overcome the lack of response to treatment using a combination of strategies. Dual inhibition of MEK and AKT has been found to result in better therapeutic effects *in vitro* and *in vivo* [32-37]. Recently, Lee et al. reported synergistic therapeutic effects of combined HSP90 and MEK inhibitors at sub-therapeutic dosages, with potent therapeutic results demonstrated in the treatment of Raf/MEK/ERK signaling pathway inhibitor resisted lung cancer with the KRAS-mutant [31].

A series of novel Raf/ERK dual inhibitors, including compound CY-9d, can induce mitochondrial apoptosis in breast cancer cells, and have previously been synergized and designed [9, 38]. Subsequent studies suggest that apoptosis induced by CY-9d is only partially dependent on the Raf/MEK/ERK pathway in breast cancer cells. This result led to a rethink of the complexity of the Raf/ERK signaling network. Additionally, a quantitative proteomics analysis of CY-9d-treated breast cancer cells revealed some potential Raf1 and ERK1 interacting proteins in breast cancer cells, such as HSP90, PAK4 and RAB2A [39-46].

The present study investigates to what extent TNBC cells bypass MEK inhibition and resist apoptosis induced by the Raf/ERK dual inhibitor CY-9d, high activation of HSP90 client proteins, and HSP90 itself. Tumor growth inhibition and apoptosis assays of CY-9d, and immunoblotting of apoptotic proteins involved in the Raf/ERK and HSP90 pathways were performed both *in vitro* and *in vivo*. A quantitative differential proteomics method was then used to elucidate the pathways potentially regulated by CY-9d. Moreover, the down-regulation of the scaffold protein IQGAP1 was determined to be related to an MEK/ERK bypassing effect and apoptosis resistance of TNBC cells. In summary, the current study indicates that the mechanism of CY-9d action in the pathways involved in Raf/MEK/ERK and Raf-HSP90 may be associated with different IQGAP1 expression levels in TNBC cells. Therefore, this suggests that the combination of CY-9d and the HSP90 inhibitor AUY-922 may be developed as an attractive therapeutic candidate for triple negative breast cancer.

## RESULTS

### The novel Raf/ERK dual inhibitor CY-9d suppresses breast cancer cell proliferation and induces apoptosis

**Figure 1:**
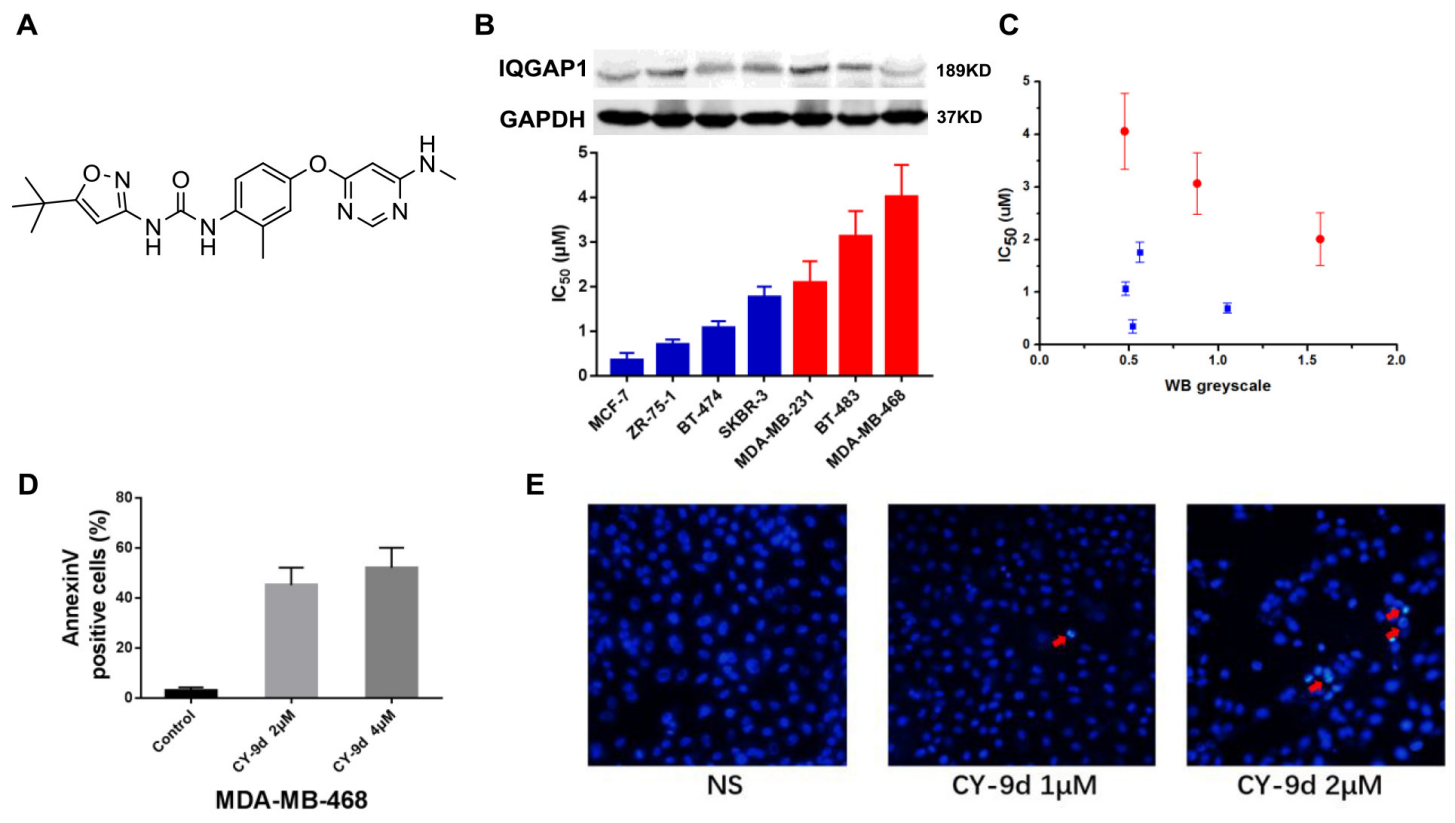
**(A)** The chemical structure of CY-9d; **(B)** The cytotoxicity of CY-9d and IQGAP protein expression level on a panel of breast cancer cells; **(C)** The correlation between WB greyscale of IQGAP1 and sensitivity of CY-9d in a panel of breast cancer cells; **(D)** The quantification of apoptotic cells after CY-9d treatment; **(E)** The cellular morphology was observed without or with CY-9d treated under fluorescent microscopy after Hoechst 33258 staining.

The chemical structure of CY-9d is shown in Figure 1A. Its cytotoxicity was screened against a panel of human breast cancer cells. The blue columns are ER/PR-positive cells, including two luminal A, one luminal B, and one HER2 subtypes, and the red columns are three TNBC cell lines. As shown in Figure 1B, the IC_50_ of CY-9d was found to be remarkably higher in TNBC cells than in ER/PR-positive cells. Moreover, the cytotoxicity of CY-9d was found to be negatively correlated with the expression level of the scaffold protein IQGAP1. The IC_50_ of CY-9d versus the relative IQGAP WB grayscale is shown in Figure 1C. An obvious linear correlation was observed, especially in TNBC cells. To further explore the detailed mechanism of CY-9d, MDA-MB-468 cells with low IQGAP levels were chosen for subsequent studies. The death subroutine of CY-9d-treated MDA-MB-468 cells was detected by flow cytometry analysis using an Annexin V/PI staining kit (Figure 1C). The percentages of Annexin V-positive apoptotic cells in the CY-9d treatment group were 43.8% ± 5.30% (2μM) and 52.7 ± 6.08% (4μM), which were found to be significantly higher than those of the control (2.5% ± 0.4%, p < 0.05) group. However, no significant difference in apoptosis between the CY-9d 2μM and 4μM groups was found. Therefore, these results suggest that obvious apoptotic cell death was initiated by CY-9d in a concentration-independent manner. Hoechst 33258 staining (Figure 1D) was used to assess morphological changes in CY-9d-treated MDA-MB-468 cells, and revealed that no apoptotic cell death was apparent after 2μM CY-9d treatment.

### IQGAP1 elicits a different therapeutic response between MCF-7 and MDA-MB-468 cells

**Figure 2:**
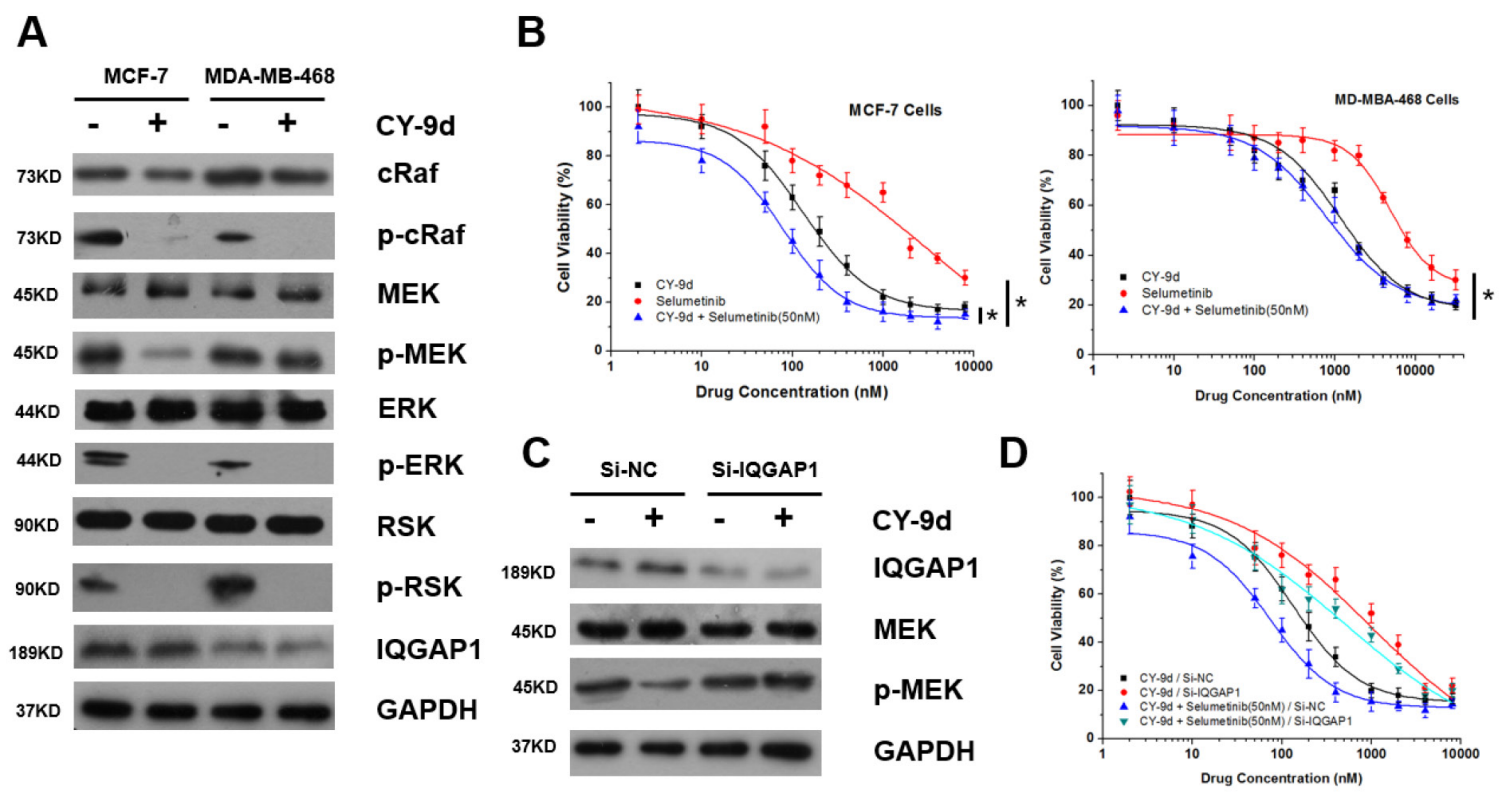
**(A)** The expression level of total and phosphorylated proteins and IQGAP1 in MCF-7 and MDA-MB-468 cells with or without CY-9d treatment; **(B)** The synergistic effects of selumetinib and CY-9d on the cytotoxicity in MCF-7 and MDA-MB-468 cells (^*^: p < 0.05); **(C)** the influence of RNAi IQGAP1 on the expression level of total and phosphorylated MEK in MCF-7 cells; **(D)** The synergistic effects of selumetinib and CY-9d on the MCF-7 cytotoxicity with or without RNAi IQGAP1.

To further validate the inhibitory effects of CY-9d on Raf/MEK/ERK pathway kinases, its suppressive capacity on the activated MAPK signal pathway kinases in MCF-7 and MDA-MB-468 cells was investigated. These cells have been shown to express different levels of IQGAP1. As shown in Figure 2A, CY-9d treatment was found to decrease the phosphorylation of cRaf, ERK and p90RSK in both cell lines. However, phosphorylated MEK levels were not found to be changed in MDA-MB-468 cells. Moreover, CY-9d treatment was not found to affect IQGAP1 or total protein levels of Raf1, MEK, ERK, p90RSK and GAPDH in either cell line. Therefore, these results suggest that CY-9d can efficiently block the Raf/MEK/ERK signaling pathway, but the detailed molecular mechanism most likely varies between MCF-7 and MDA-MB-468 cells.

Based on the varying changes in the phosphorylation level of MEK after CY-9d treatment in MCF-7 and MDA-MB-468 cells, the synergistic effects of CY-9d and the MEK inhibitor Selumetinib were tested in both cell lines (Figure 2B). As expected, the addition of Selumetinib at a sub-therapeutic concentration was found to significantly increase the cytotoxicity of CY-9d in MCF-7 cells but not MDA-MB-468 cells. To further assess whether the independence of MEK phosphorylation in MDA-MB-468 cells was related to the expression of IQGAP1, an RNA interference (RNAi) experiment using small interfering RNA (siRNA) targeting IQGAP1 was performed in MCF-7 cells (Figure 2C). After IQGAP1 knockdown by si-IQGAP1, the CY-9d-induced down-regulation of p-MEK was found to disappear. In addition, when IQGAP1 was knocked down in MCF-7 cells, the cytotoxicity of CY-9d and the synergistic effects of selumetinib and CY-9d were found to decrease. Therefore, IQGAP1 level may be important both in the therapeutic response to CY-9d and in the Raf-MEK-ERK interactions in breast cancer cells. Finally, the crosstalk between Raf and ERK may be complicated, except for the routine Raf/MEK/ERK cascade.

### Quantitative proteomics analysis of CY-9d-induced apoptotic pathways

iTRAQ and MS/MS analysis of RAF or ERK inhibitor-treated MDA-MB-468 cells were performed. These cells were dissolved in a lysis buffer in the presence of a protease inhibitor (Sigma). The lysate was centrifuged for 1 hour at 15°C, and the supernatant was stored at −80°C until further use. Protein quantitation was performed using an RCDC Protein Assay Kit (Bio-Rad). iTRAQ labeling was performed using an iTRAQ Reagent4-Plex kit (AB SCIEX) according to manufacturer protocols with minor modifications. HCT116-Control and HCT116-CacyBP OE whole-cell lysates were labeled with iTRAQ labeling reagent 114 and 115 for the control MCF-7 and compound 9d-treated MCF-7 whole-cell lysates, respectively. iTRAQ labeling reagent 113 was used to label control MCF-7 cells, and iTRAQ labeling reagents 114 and 117 were used to labeled ERK inhibitor-treated MCF-7 cells. After 2D LC and tandem mass spectrometry analysis, protein identification and relative iTRAQ quantification were performed with ProteinPilot™ Software 4.2 (AB SCIEX) using the Paragon™ algorithm for peptide identification. This was further processed using the ProGroup™ algorithm with isoform-specific quantification, which was adopted to trace the differences between the expressions of various isoforms. Results with iTRAQ ratio cutoff values of 1.2 and 0.8 for fold-change and number cutoff values of 3 for quantifiable peptides for protein abundance were accepted. Moreover, the results for 114 and 117 were only adopted when the changes in their expression levels were found to follow the same trend.

**Figure 3:**
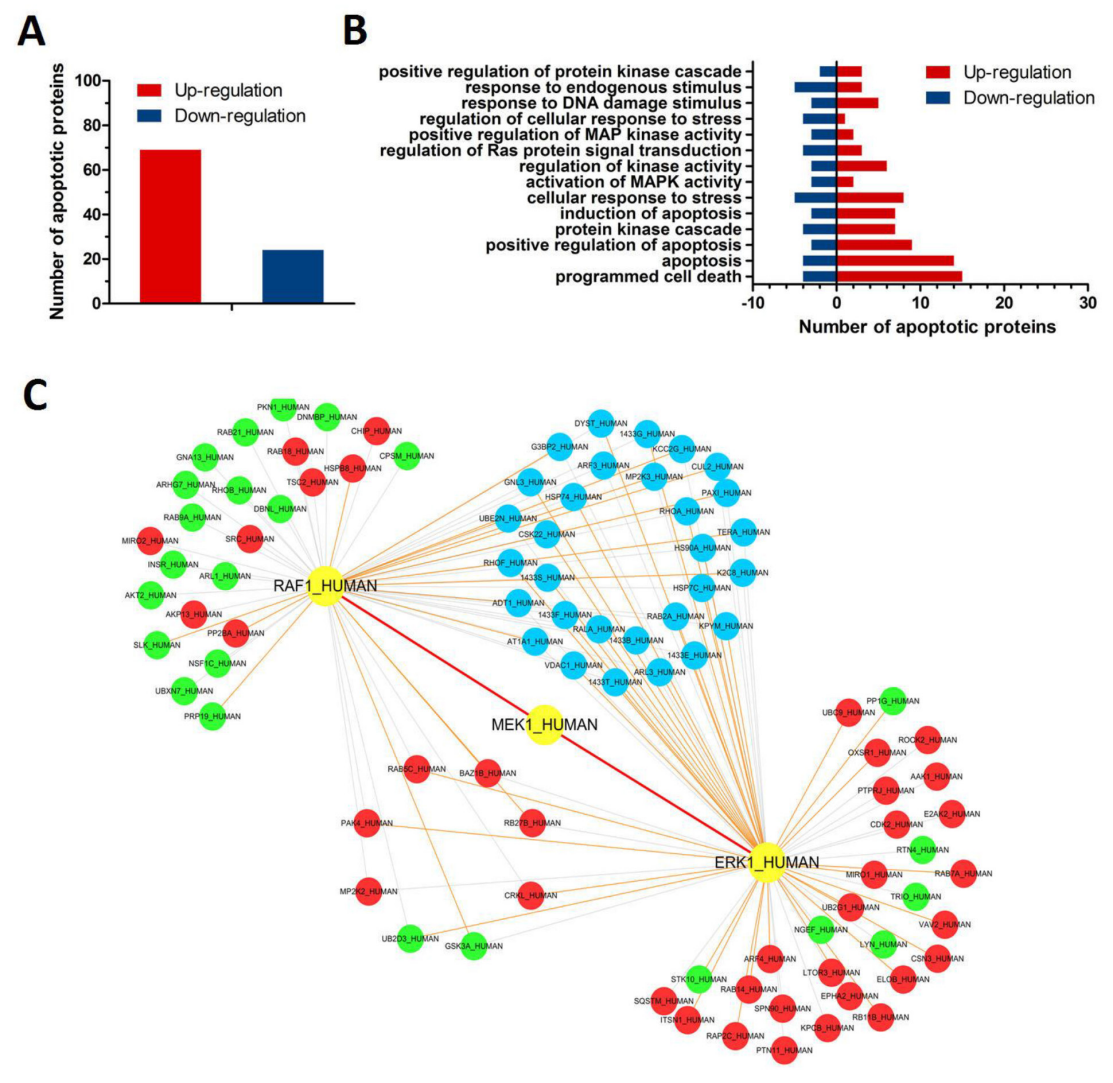
Proteomics analyses of CY-9dinduced apoptosis in MDA-MB-468 breast cancer cells **(A)** Different protein levels (upregulation or downregulation) between control and 9d-treated MDA-MB-468 cells; **(B)** Apoptosis-related enrichment analyses in CY-9dtreated MDA-MB-468 cells; **(C)** Proteomics-based identification of novel Raf1-ERK apoptotic pathways in CY-9dtreated MDA-MB-468 cells.

To build the RAF/MEK/ERK kinase protein–protein interaction (PPI) network, diverse sets of biological evidence from seven online databases was collected. Different PPIs were collected from the Database of Interacting Proteins (DIP), Biomolecular Object Network Databank (BOND), Human Protein Reference Database (HPRD), HomoMINT, IntAct, BioGRID and PrePPI. Moreover, the RAF/MEK/ERK subnetwork from the PPI network was extracted based on ITRAQ-based proteomics results (Figure 3). Finally, some potential Raf1 and ERK1 interacting proteins in breast cancer cells were detected, such as HSP90, PAK4 and RAB2A.

### HSP90 inhibitor AUY922 sensitizes MDA-MB-468 cells with resistance to CY-9d

**Figure 4:**
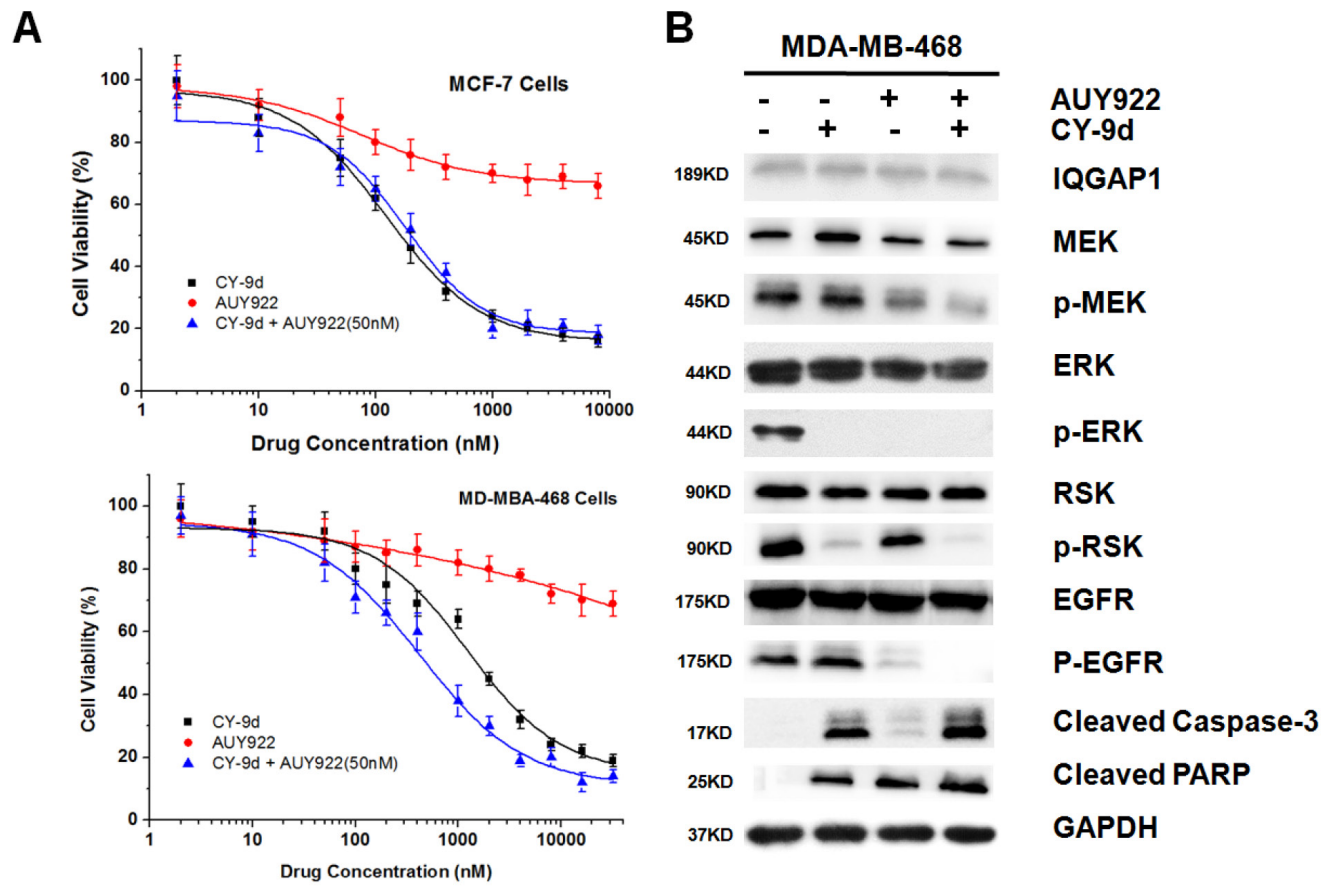
CY-9d and AUY922 synergize in inducing apoptosis **(A)** Synergistic effect of the combination of CY-9d and AUY922. Proliferation assays were performed in MCF-7 and MDA-MB-468 cells treated with 50 nM AUY-922 plus increasing concentrations of CY-9d; **(B)** Effects of the combination of AUY922and CY-9d on downstream signaling and apoptosis marker, GAPDH was included as a loading control.

The down-regulation of IQGAP1 was found to disrupt the routine Raf/MEK/ERK cascade, and HSP90 was detected as a novel interactor between cRaf and ERK1. The two cell lines to the HSP90 inhibitor NVP-AUY922 and CY-9d were exposed to determine to what extent the HSP90 inhibitor could overcome the CY-9d resistance of TNBC cells (Figure 4A). In these studies, the combination of Cy-9d with 50 nM AUY922 was found to show distinct synergy in MDA-MB-468 cells but not MCF-7 cells. Furthermore, western blotting (WB) analysis of IQGAP1, MEK, p-MEK, ERK, p-ERK, RSK, p-RSK, EGFR, p-EGFR, cleaved caspase-3, and PARP in the treated MDA-MB-468 cells was performed (Figure 4B). Both CY-9d and AUY922 were not found to influence the expression of IQGAP1, RSK and EGFR, whereas the expression of total MEK, p-MEK and total ERK was found to be reduced by AUY-922. Moreover, the phosphorylation of EGFR, a typical HSP90 client protein, was found to be inhibited by AUY-922 but not by CY-9d. The degradation of caspase-3 and PARP was found to be enhanced in the combination group, suggesting synergistic effects of AUY-922 and CY-9d in apoptosis induction.

### CY-9d potently inhibits tumor growth and induces apoptosis in xenograft models

**Figure 5:**
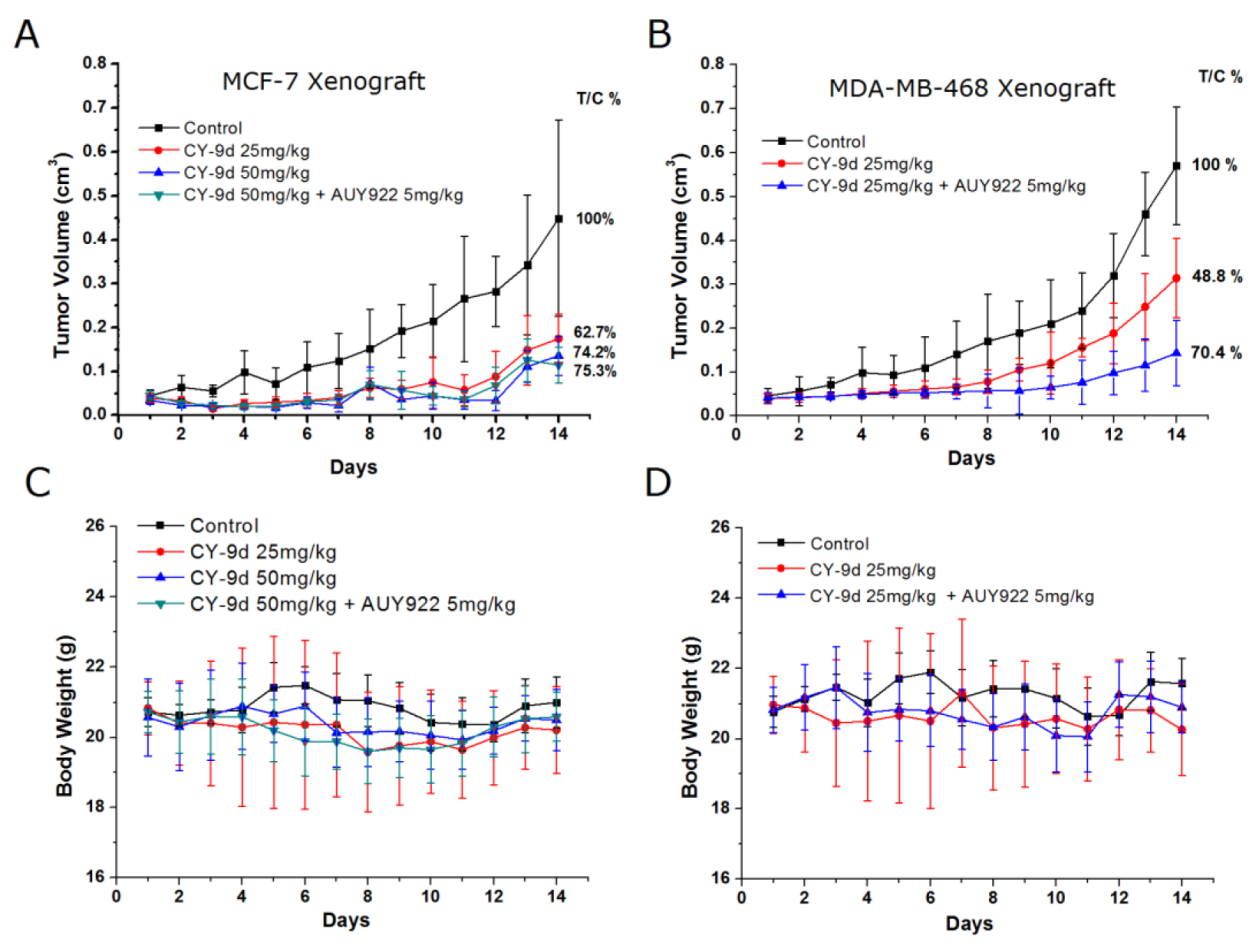
AUY922 combined with CY-9d suppressed breast cancer growth *in vivo* Mice were injected subcutaneously with MCF-7 and MDA-MB-468 cells, respectively. The tumor volume **(A and B)** and relative body weight **(C and D)** were shown with the mean ± SD of six mice in each group. Mice were treated with vehicle, CY-9d at 25 or 50 mg/kg· day for 14 days, or combined with AUY922 at 5 mg/kg mg/kg· day for 14 days.

**Figure 6:**
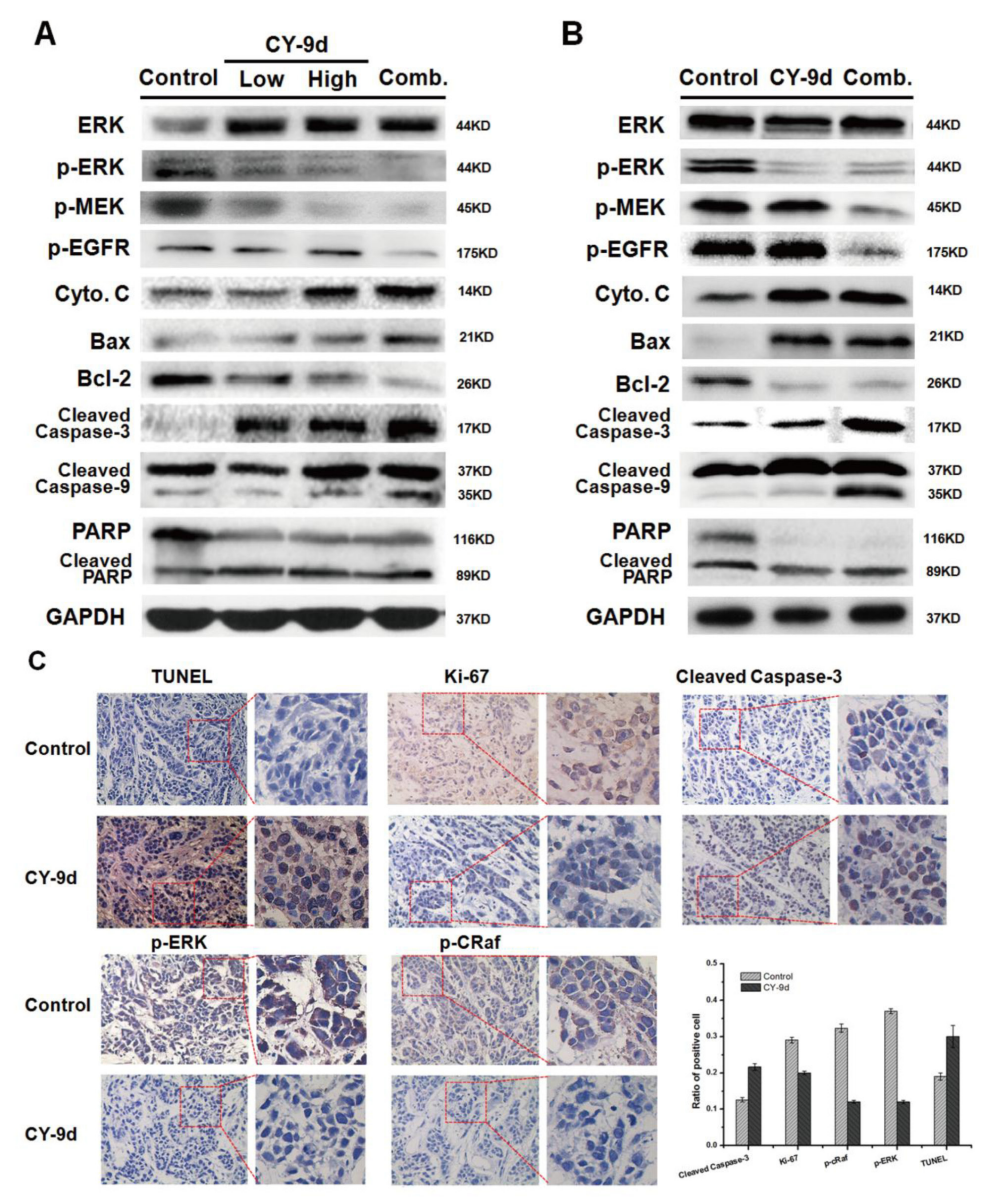
Western blotting and immunohistochemically analysis of tumor tissues from vehicle, CY-9d and CY-9d-AUY922 combined treated mice **(A)**. The western blotting analysis of MCF-7 xenograft tumor issues for expression of ERK, p-ERK, p-MEK, p-EGFR, Cytochrome C, Bax, Bcl-2, PARP, cleaved caspase-3 and 9, GAPDH was measured as loading control; **(B)**. The western blotting analysis of MDA-MB-468 xenograft tumor issues for expression of ERK, p-ERK, p-MEK, p-EGFR, Cytochrome C, Bax, Bcl-2, PARP, cleaved caspase-3 and 9, GAPDH was measured as loading control; **(C)**. The TUNEL apoptosis assay, Ki-67 cell proliferation assay and expression of cleaved Caspase-3, p-ERK and p-cRaf in representative MDA-MB-468 xenograft tumor sections of mice after vehicle and CY-9d treated were measured by immunohistochemically analysis and quantification of immunoblot, Every experiment was performed from 3 independent duplicates.

To what extent CY-9d demonstrates similar inhibitory effects on the Raf/MEK/ERK and apoptosis pathways was determined both *in vivo* and in combination with AUY-922. MCF-7 and MDA-MB-468 human breast cancer xenograft models were established to evaluate the efficacy of CY-9d. Mean tumor data after 14 days of treatment are presented in Figures 5 and 6. Both CY-9d by itself and in combination with AUY-922 was found to exhibit measurable antitumor activity at the three doses tested, and the lowest rate of tumor inhibition was 48.8%. Moreover, the CY-9d-treated group was not found to exhibit a dose-dependent decrease in tumor volume compared with the vehicle control (Figure 5A), with total tumor growth inhibition ratios (T/C) in the CY-9d 25 mg/kg and 50 mg/kg groups of 62.7% and 74.2%, respectively. As expected, no difference between the CY-9d group and the CY-9d/AUY-922 combination group was found. Therefore, CY-9d was found to be effective in reducing the growth of MCF-7 tumors in a breast cancer xenograft model in mice. By contrast, treatment with CY-9d alone was found to only moderately suppress the growth of MDA-MB-468 xenograft tumors. Finally, the combination of 5 mg/kg AUY-922 and 25 mg/kg CY-9d was found to efficiently inhibit tumor progression, with T/C values increasing from 48.8 % to 70.4 %.

Furthermore, to confirm whether the molecular mechanism of CY-9d and the HSP90 bypass pathway were valid *in vivo*, the changes in the Raf/MEK/ERK pathway, HSP90 client proteins and apoptosis-related proteins were measured using western blotting and immunohistochemical methods. As shown in Figure 6A, in the MCF-7 xenograft models, the phosphorylation of ERK and MEK was shown to be almost completely inhibited in the three treated groups, and p-EGFR was found to be suppressed only in the combination group. Cytochrome C was found to be activated in the CY-9d 50mg/kg group and the combination group, and treatment with CY-9d 25mg/kg was not found to activate cytochrome C. For other apoptosis-related proteins, such as Bax, Bcl-2, PARP, and cleaved caspase-3 and 9, the increase in CY-9d dosage or combination with AUY-922 was not found to increase tumor growth inhibition or apoptosis-inducing effects. By contrast, in the MDA-MB-468 xenograft models, the addition of AUY-922 was found to suppress the phosphorylation of EFGR and MEK and induce more cleaved caspase-3 and 9, suggesting that the combination of CY-9d and AUY-922 enhanced the apoptotic death of MDA-MB-468 cells *in vivo*. Moreover, the expression levels of p-ERK, p-cRaf and cleaved caspase-3 in MDA-MB-468 xenograft tumor tissues and CY-9d-treated groups were measured by immunohistochemical (IHC) analysis. Both p-ERK and p-cRaf were found to be reduced in the CY-9d-treated group, and cleaved caspase-3 was found to be elevated. Ki-67 is a proliferation marker with a prognostic and predictive potential in breast cancer. The results of the IHC analysis and statistical analysis are presented in Figure 6C. A significant reduction in the expression of Ki-67 was observed upon CY-9d treatment. Histological assessment of apoptosis by TUNEL staining revealed that TUNEL-positive apoptotic nuclei were significantly increased by CY-9d.

## DISCUSSION

TNBC is highly aggressive, and effective chemical or radiative therapeutics are lacking. Due to the relative genetic complexity of TNBC, targeted therapies are also limited. Although TNBC cells are heterogeneous, they share some signaling pathways, suggesting that this type of malignancy may be highly dependent on the expression of a certain subset of genes. A novel series of Raf/ERK dual inhibitors with potent therapeutic effects in breast cancers both *in vitro* and *in vivo* were previously discovered. The low expression of IQGAP1 was found to mediate the resistance of TNBC to Raf/ERK inhibition. Additionally, the interactions between IQGAP1 and the Raf/MEK/ERK cascade were found to be potential therapeutic targets for this subtype of breast cancer [47-49]. After IQGAP1 knockdown by siRNA, MCF-7 cells were found to be resistant to the Raf/ERK dual inhibitor CY-9d, and MEK inhibition and CY-9d no longer showed synergistic effects. Similarly, the combination of the MET inhibitor Selumetinib and CY-9d was not found to synergistically reduce TNBC cell proliferation. Moreover, IQGAP1 protein level was found to be negatively correlated with the response to CY-9d in both ER/PR-positive breast cancer cells and TNBC cells. IQGAP1 functions as a scaffold protein in the formation of MAPK signaling complexes and regulates MAPK signaling by scaffolding several MAPK components, including Raf, MEK and ERK [50]. The scaffold function of IQGAP1 was found to promote the activation of the ERK pathway, which was found to influence various cellular processes.

**Figure 7:**
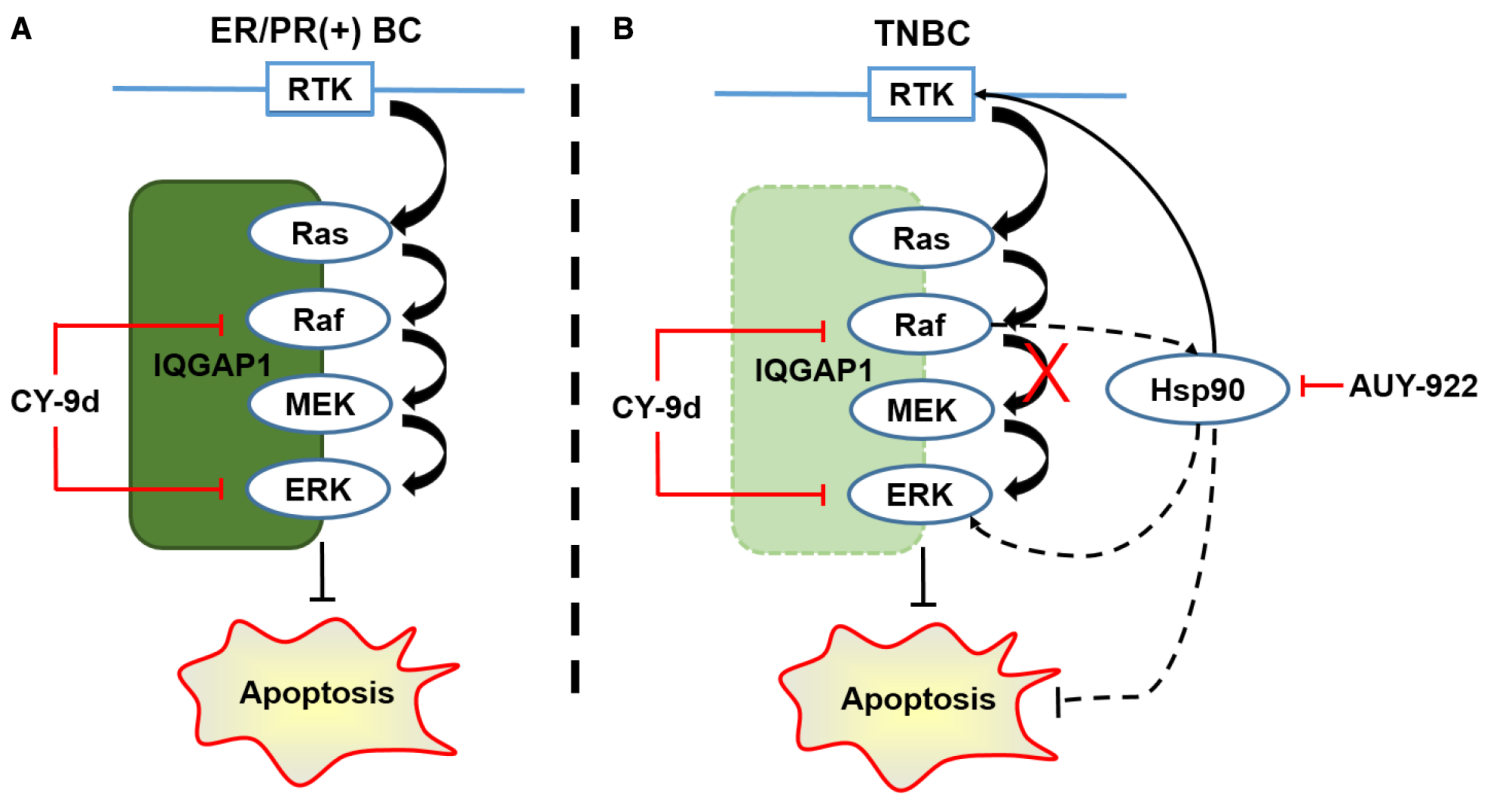
The probably mechanisms of CY-9d and its synergistic effect with AUY-922 in breast cancer cells **(A)** Canonical Raf/MEK/ERK cascade in ER/PR positive breast cancer with IQGAP1 as scaffold; **(B)** HSP90 as a bypass pathway to the down-regulated IQGAP1 induced Raf/MEK/ERK-independent of CY-9d via client proteins including EGFR in TNBC.

Quantitative proteomics analysis was employed to identify the differentially expressed proteins after CY-9d treatment. Differentially expressed proteins were assigned to 14 functional synaptic protein groups. Enrichment was only considered relevant when over-represented functional groups contained at least 5 proteins. In addition, functional enrichment was determined using the DAVID functional annotation tool. The functional categories used were GO terms related to Biological Process (BP). Apoptosis-related proteins were detected and used as a background set. The iTRAQ results suggested the presence of some potential Raf1-ERK1-interacting proteins. Among these proteins, heat shock protein 90 (HSP90) was predicted as a bypass pathway for IQGAP1-loss-induced independence of the Raf/MEK/ERK pathway in TNBC cells (Figure 7). HSP90 is a highly conserved chaperone protein involved in the stabilization and maturation of many client oncoproteins, such as epidermal growth factor receptor (EGFR), cRaf, ERK, Cdk4/6, Akt, Bcr-Abl, and p53. These client oncoproteins are essential in oncogenesis, cancer cell proliferation and apoptosis resistance [51-55]. As expected, the combination of CY-9d and the HSP90 inhibitor AUY-922 were found to exhibit potent synergistic effects in the cytotoxicity assay of TNBC cells but not ER/PR-positive cells. Western blotting analysis of proteins in the Raf/MEK/ERK pathway, HSP90 client proteins and apoptosis-related proteins indicated that the addition of AUY-922 enhanced the apoptosis-inducing capacity of CY-9d in TNBC cells. To further validate the anti-tumor potency and mechanism of CY-9d in ER/PR-positive and triple negative breast cancers, the *in vivo* tumor growth suppression capacities of CY-9d or the CY-9d/AUY-922 combination in MCF-7 and MDA-MB-468 xenograft models were determined. Consistent with previous research, CY-9d was found to potently inhibit MCF-7 cell growth *in vitro* and *in vivo*. In addition, no synergistic effects were observed in the combined group, suggesting that the HSP90 bypass pathway may not activate in MCF-7 cells. Opposing results were obtained in the MDA-MB-468 models based on tumor growth curve, western blotting and immunohistochemical analysis results. The WB and IHC results showed that the phosphorylation of Raf, MEK, ERK and EGFR, a representative HSP90 client protein, was suppressed by the combination of CY-9d and AUY-922. Finally, the apoptosis markers TUNEL, PARP, cleaved caspase-3 and 9 were all found to be activated, indicating that the dual inhibition of the Raf/MEK/ERK and HSP90 pathways with sub-therapeutic doses may provide a novel therapeutic strategy for treating TNBC.

## MATERIALS AND METHODS

### Cell culture and reagents

The human breast cancer cell lines MDA-MB-231, MDA-MB-468, MCF-7, BT-474, BT-483, SKBR-3, ZR-75-1, Cells were incubated under sterile conditions at 37°C and were maintained in a humidified atmosphere 5% (vol/vol) CO_2_ with RPMI-1640 or DMEM medium containing 10% fetal bovine serum (GIBCO, Waltham, MA, USA). NVP-AUY922 (Luminespid) and selumetinib (AZD6244) were acquired from Selleck Chemicals (Houston, TX, USA), dissolved in DMSO to a final concentration of 10 mM, and stored at −20 °C.

MTT assay was performed to evaluate the cellular proliferation inhibitory activities of CY-9d by a panel of breast cancer cells. In general, cells were seeded into 96-well plates and treated with a series of concentration of test drugs for 24h. The MTT reagent (5mg/ml) was added per well for 3h at 37°C. After that, the MTT was removed and 150 μl DMSO was added to dissolve the formazan crystals. Then, optical density (OD) was measured at 570 nm of the solution. The control group consisted of untreated cells. The percentage of cell viability averaged from three individual experiments.

### Apoptosis assay

Apoptosis induction assay processed by FACS in MDA-MB-468 cells treated with CY-9d. The flow cytometric analysis was used to identify different micelles apoptosis inducing effect. MDA-MB-468 treated with CY-9d or saline (control) were gently trypsinized without EDTA and centrifuged at 2000g for 5 minutes. Then, the harvested cells were washed with 1.0 ml ice cold PBS and re-suspended in 500 μl 1×binding buffer, and incubated with 5 μl of Annexin V-FITC and 5 μl of propidium iodide (PI) for 15 min at room temperature. Followed by FCM (BD FACS Calibur, BD, USA) using the FL1 channel for Annexin V-FITC and the FL2 channel of PI. Both early apoptotic (Annexin V+/PI-) and late apoptotic (Annexin V+/PI+) cells are included in the cell assay of apoptosis.

### Quantitative proteomics analysis

The iTRAQ and MS/MS analysis were performed in CY-9d-treated MD-MBA-468 cells. Briefly, cells were dissolved in lysis buffer in presence of protease inhibitor (Sigma). The lysate was centrifuged for 1 h at 15°C and the supernatant was stored at −80°C for further use. Protein quantitation was performed using RCDC Protein Assay Kit (Bio-Rad). iTRAQ labeling was carried out using iTRAQ Reagent4-Plex kit (AB SCIEX) based on the manufacturer’s protocol with minor modifications. A pair of MD-MBA-468-Control and MD-MBA-468-CacyBP OE whole cell lysates was labeled with iTRAQ labeling reagent 114 and 115for the pair of control MD-MBA-468 and CY-9d-treated MD-MBA-468 whole cell lysate, respectively.113 iTRAQ labeling reagents were used to labeled Control MD-MBA-468, 114 and 117iTRAQ labeling reagents were used to labeled CY-9d treated MD-MBA-468, respectively. After 2D LC analysis and tandem mass spectrometry analysis, protein identification and relative iTRAQ quantification were performed with ProteinPilot™ Software 4.2 (AB SCIEX) using the Paragon™ algorithm for the peptide identification, which was further processed by ProGroup™ algorithm where isoform-specific quantification was adopted to trace the differences between expressions of various isoforms. Results with iTRAQ ratio cutoff values of 1.2 and 0.8 for fold-change and number cutoff values of 3 for quantifiable peptides for in protein abundance were accepted. Moreover, we only adoptedthe results of 114 and117 when their expression levels were changed at the same trend.

### Proteomics-based PPI network construction

To identify of protein-protein interactions involved in RAF/MEK/ERK, we collected experimentally supported interaction pairs from seven online protein interaction databases, inducing the Database of Interacting Proteins (DIP), Biomolecular Object Network Databank (BOND), Human Protein Reference Database (HPRD), Biological General Repository for Interaction (BioGRID), HomoMINT, IntAct and PrePPI. After removing duplicate interactions, we extracted the binary RAF/MEK/ERK PPI network based on the iTRAQ-based proteomics results.

### Western blot and RNA interference

The different concentrations of drug treated cells were harvested and washed with cold 1×PBS. Total cell lysates were prepared in lysis RIPA buffer (Invitrogen, CA, USA) on ice for 30 min, followed by centrifugation at 13000 rpm for 30 min at 4°C. After collecting supernatant, protein concentration was determined by a bicinchoninic acid protein assay kit (Thermo, USA). The protein was resolved on a 10-15% SDS-polyacrylamide gel, electro blotted onto nitrocellulose membranes, and then incubated with proper primary antibodies which were purchased from Cell Signaling Technology or Santa Cruz Biotechnology and secondary antibodies before visualization by chemiluminescence Kit (Millpore, USA). Though the use of small interfering RNAs (siRNAs) against human IQGAP1 on RNA interferences (RNAi). Bought siRNAs from RuiBo Co. Ltd. Use of Lipofectamine 2000, according to the manufacturer’s instructions, with 100 nm final concentration of siRNA transfection MDA-MB-468 cells. Transfection of cells for subsequent experiments after 24 hours.

### Xenograft tumor model, histologic assessment and primary safety evaluation

*In vivo* activity and toxicity of CY-9d were carried out according to the Guidelines for the Care and Use of Laboratory Animals that were approved by the by the Committee of Ethics of Animal Experimentation of Sichuan University. Female specific pathogen-free nude mice (6∼8-week-old), provided by Huafukang Biotechnology Co., Ltd, and were randomly grouped by weighed and coded (n=8). After about week adaptation, the mice were grafted s.c. into the dorsal flank with 0.1 ml of phosphate buffer containing 2×10^6^ MDA-MB-468 cells. When the tumors grew to a size of approximate leg diameter of 6 mm, the mice were initially treated and orally administered with CY-9d, CY-9d combined with AUY-922 or saline on days 1-14, and were monitored on a daily basis during treatment (tumor volume and body weights). On the last day, the animals were sacrificed, tumors and main organs were isolated and weighed. The tumor tissue fixed in 4% paraformaldehyde in PBS for immunohistochemistry analysis and the remaining tissue was stored at -80^o^C for Western blotting. The various organs were sectioned, stained with H&E, TUNEL, p-ERK, Ki-67, p-cRaf and caspase 3 antibodies as the previous studies stated.

## CONCLUSION

In summary, the novel Raf/ERK dual inhibitor CY-9d potently inhibited the proliferation of a panel of breast cancer cells and induced mitochondrial apoptosis. In TNBC cells, down-regulation of IQGAP1 has been found to reduce the routine Raf/MEK/ERK cascade and lead to resistance to CY-9d. Moreover, a quantitative proteomics analysis has been used to explore possible apoptosis-related proteins regulated by CY-9d induced signaling pathways. HSP90 has been found to be a potential bypass pathway in MDA-MB-468 cells. Furthermore, by inducing mitochondrial apoptosis, the combination of CY-9d and the HSP90 inhibitor AUY-922 has been found to synergistically suppress tumor growth in TNBC xenograft models but not ER/PR-positive breast cancer models. Taken together, these integrated proteomic *in vitro* and *in vivo* studies indicate that the synthetic Raf1/ERK dual inhibitor CY-9d and its combined administration with an HSP90 inhibitor may be potential therapeutic strategies for the treatment of triple negative breast cancer.
